# Proximity to Golf Courses and Risk of Parkinson Disease

**DOI:** 10.1001/jamanetworkopen.2025.9198

**Published:** 2025-05-08

**Authors:** Brittany Krzyzanowski, Aidan F. Mullan, E. Ray Dorsey, Sai Shivani Chirag, Pierpaolo Turcano, Emanuele Camerucci, James H. Bower, Rodolfo Savica

**Affiliations:** 1 Department of Neurology, Barrow Neurological Institute, Phoenix, Arizona; 2 Department of Neurology, Mayo Clinic, Rochester, Minnesota; 3 Department of Neurology, Center for Health + Technology, University of Rochester Medical Center, Rochester, New York; 4 Department of Neurology, University of Kansas Medical Center, Kansas City, Kansas; 5 Department of Quantitative Health Sciences, Mayo Clinic, Rochester, Minnesota

## Abstract

**Importance:**

The role of pesticide exposure from golf courses in Parkinson disease (PD) risk remains unclear.

**Objective:**

To assess whether proximity to golf courses is associated with increased PD risk and to use information on groundwater vulnerability and municipal well locations to investigate drinking water contamination as a potential route of exposure.

**Design, Setting, and Participants:**

This case-control study included patients with incident PD and matched controls from the Rochester Epidemiology Project from 1991 to 2015. Data were analyzed between June and August 2024.

**Exposures:**

Distance to golf courses, living in water service areas with a golf course, living in water service areas in vulnerable groundwater regions, living in water service areas with shallow municipal wells, and living in water service areas with a municipal well on a golf course.

**Main Outcome and Measures:**

Risk of incident PD. All models adjusted for age, sex, race and ethnicity, year of index, median household income, and urban or rural category.

**Results:**

A total of 419 incident PD cases were identified (median [IQR] age, 73 [65-80] years; 257 male [61.3%]) with 5113 matched controls (median [IQR] age, 72 [65-79] years; 3043 male [59.5%]; 4504 White [88.1%]). After adjusting for patient demographics and neighborhood characteristics, living within 1 mile of a golf course was associated with 126% increased odds of developing PD compared with individuals living more than 6 miles away from a golf course (adjusted odds ratio [aOR], 2.26; 95% CI, 1.09-4.70). Individuals living within water service areas with a golf course had nearly double the odds of PD compared with individuals in water service areas without golf courses (aOR, 1.96; 95% CI, 1.20-3.23) and 49% greater odds compared with individuals with private wells (aOR, 1.49; 95% CI, 1.05-2.13). Additionally, individuals living in water service areas with a golf course in vulnerable groundwater regions had 82% greater odds of developing PD compared with those in nonvulnerable groundwater regions (aOR, 1.82; 95% CI, 1.09-3.03).

**Conclusions and Relevance:**

In this population-based case-control study, the greatest risk of PD was found within 1 to 3 miles of a golf course and risk generally decreased with distance. Associations with the largest effect sizes were in water service areas with a golf course and in vulnerable ground water regions.

## Key Points

**Question:**

Does living within proximity to a golf course affect the risk of Parkinson disease (PD)?

**Findings:**

This case-control study found the greatest risk of PD within 1 to 3 miles of a golf course, and that this risk generally decreased with distance. Effect sizes were largest in water service areas with a golf course in vulnerable groundwater regions.

**Meaning:**

These findings suggest that pesticides applied to golf courses may play a role in the incidence PD for nearby residents.

## Teaser

This case-control of older US adults examines risk of Parkinson Disease by proximity of residence to a golf course.

## Introduction

Parkinson disease (PD) is a neurodegenerative disease likely caused by a complex interaction between environmental factors^1,2,3^ and genetic predisposition.^4,5,6^ Among the environmental risk factors, pesticide exposure has been linked to increased risk of PD.^7,8,9,10,11,12^ Golf courses are often treated with pesticides to maintain the aesthetic standards for putting greens and fairways,^13^ and in the US, pesticide application to golf courses can be up to 15 times higher compared with countries in Europe.^14^ One report anecdotally implicated proximity to golf courses as a risk factor for PD.^15^ Furthermore, pesticides applied to golf courses can leach into the groundwater and contaminate drinking water.^16,17^

Despite the possible risks, research on pesticide exposure from golf courses and PD remains sparce. For this reason, we conducted a population-based study using data from the Rochester Epidemiology Project (REP) medical records–linkage system to explore the association between incident PD and proximity to 139 golf courses within a 16 119 square mile multicounty study region in southern Minnesota and Western Wisconsin. We hypothesized that individuals with addresses history in proximity to golf courses would have greater risk of incident PD compared with those who lived further away. Additionally, we hypothesized that we would observe greater risk of PD in individuals living within water service areas: (1) with a golf course, (2) on vulnerable groundwater regions, or (3) with shallow municipal wells.

## Methods

A review waiver and exemption of informed consent was granted by the Mayo Clinic institutional reviewer board, and use of medical records had Minnesota research authorization. Results conform to Strengthening the Reporting of Observational Studies in Epidemiology (STROBE) reporting guideline.

### Assessment of Parkinson Disease

We identified patients with PD in Olmstead County from 1991 to 2015 using codes from the *International Classification of Diseases, Ninth Revision (ICD-9)* (332.0, 333.0, 331.82) and *International Statistical Classification of Diseases and Related Health Problems, Tenth Revision (ICD-10)* (G20, G21, G23.1, G23.2, G31.83) within the REP medical records–linkage system.^18^ The medical records of all patients identified by *ICD* codes were reviewed by a movement disorder specialist (R.S.) to confirm the diagnosis and determine date of motor symptom onset. Both motor and cognitive symptoms were reviewed during diagnosis,^18^ and concordance between clinical and pathological diagnoses was confirmed in a subset of patients who underwent brain autopsy.^19^ Although PD cases were required to be living in Olmsted County at symptom onset, they were not required to have lived in Olmsted County before that date. Controls were identified from the 27-county REP study region in Minnesota and Wisconsin.^20^ Controls were matched to PD cases on sex and age at index date (date of PD symptom onset for the matched case) using a 20:1 match because we expected controls to have less residency information compared with PD cases given the prodromal period and the arbitrary nature of the index date. All controls were required to not have any *ICD* codes for PD prior to the index date or up to 5 years following.

### Proximity to Golf Courses

**Figure 1.**
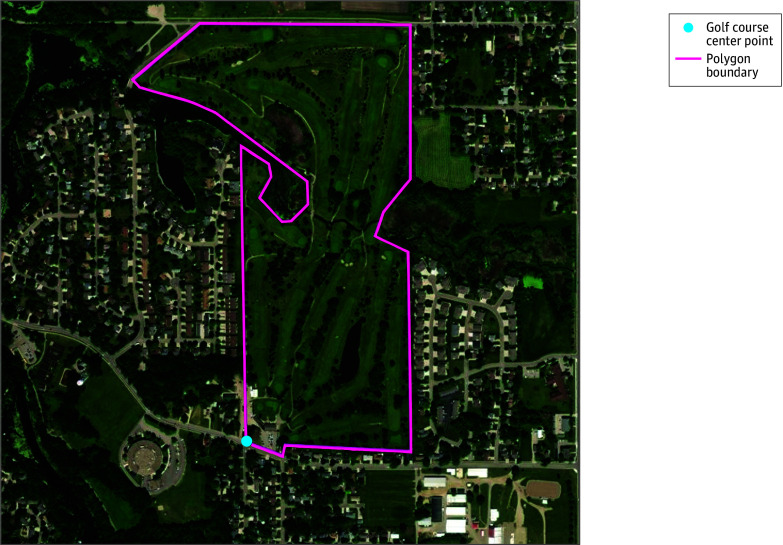
Overhead Satellite Image of Golf Course Map With Polygon Boundaries Golf course center points were converted to polygon boundaries outlining the course using manual digitization and satellite imagery for the year 2022 from ESRI, Maxar, and Earthstar Geographics. Imagery from Esri, Maxar, Earthstar Geographics, and the GIS User Community, 2024.

Data on golf course locations in 2013 were collected from Esri Business Analyst^21^ for 139 golf courses within the 27-county REP study region. We included golf courses beyond the perimeter of the study area so that we could calculate accurate distance values for those living at the edge of our study region. Golf course center points were converted to polygon boundaries outlining the course using manual digitization and satellite imagery in ArcPro version 10.0 (Environmental Systems Research Institute) (Figure 1). The distance in miles to the edge of the nearest golf course specific to each PD patient and control was identified based on the latitude and longitude of their home address of residency (eFigure 1 in Supplement 1). The home address 2 or 3 years prior to PD symptom onset (for individuals with PD) or index date (controls) was used to calculate distance to allow for a delay between potential environmental exposure and the development of PD motor symptoms.

### Water Service Area, Groundwater Vulnerability, and Municipal Wells

Data on 224 water service areas in our 27-county REP study area were collected from the US Geological Survey. Water service area data were categorized as either service areas where tap water comes from (1) groundwater resources, (2) surface water resources, or (3) private wells, wherein everyone living within the same water service area is often distributed water from the same shared or community drinking water resource (groundwater or surface water) and everyone outside of water service areas gets their drinking water from private wells. Water service areas were also categorized as either containing a golf course or not containing a golf course. Additionally, we also obtained high resolution data on groundwater vulnerability for the state of Minnesota from the Minnesota Department of Agriculture wherein groundwater vulnerability was defined as regions with coarse textured soils, shallow bedrock, or karst geology. Groundwater vulnerability is relevant to drinking water sourced from groundwater and private wells. We assigned water service areas as being either within a vulnerable groundwater region or within a nonvulnerable groundwater region (eFigure 2 in Supplement 1). Finally, we obtained data on the locations of 711 municipal wells in southeastern Minnesota from the Minnesota Geospatial Information Office and assigned water service areas as having either no municipal wells on golf courses or at least 1 municipal well on a golf course; and water service areas containing deep municipal wells (100 ft or deeper) or at least 1 shallow municipal well (less than 100 ft deep).

### Statistical Analysis

In our case-control study, we modeled the exposure (distance to nearest golf course) as a continuous variable (in miles). The association between risk of PD and proximity to nearest golf course was evaluated using piecewise linear splines to account for differences in the association at closer or farther distances.^22^ The placement of an intersection knot between linear splines was determined using bootstrap resampling^23^ optimized on the out-of-bag area under the receiver operating characteristics (ROC) curve. Candidate knots were considered at all half-mile intervals. Additionally, we also modeled the distance to the nearest golf course as a categorical variable (less than 1, 1 to 2, 2 to 3, 3 to 6, and over 6 miles) to allow our results to be more easily compared with other studies.

Logistic regression was used with PD as the outcome and distance to nearest golf course as the exposure variable, adjusting for age, sex, race, ethnicity, year of index, block group–level median household income in 2010,^24^ residency Rural Urban Commuting Area (RUCA) from 2010,^25^ and health care utilization. RUCA designation was categorized as urban (coded as 1), suburban (2 to 6), and rural (7 to 10). Health care utilization prior to the index date was defined as the mean number of days per year with at least *ICD-9* or *ICD-10* code recorded in the medical record, averaged over the 5 years prior to the index date. Results were reported as adjusted odds ratios (aORs) with 95% CIs. Secondary analysis evaluated the association between water service area characteristics and risk of developing PD. The primary water service area characteristics were type of water service area (groundwater with a golf course, groundwater without a golf course, and private well), groundwater vulnerability (vulnerable groundwater with a golf course, vulnerable groundwater without a golf course, nonvulnerable groundwater with a golf course, and nonvulnerable groundwater without a golf course), minimum well depth in the water service area (shallow [less than 100 ft] or deep [100 ft or deeper]), and location of municipal wells (at least 1 well on a golf course vs no wells on a golf course). As a sensitivity analysis, we repeated our analysis after stratifying by RUCA (comparing our results with results of those living in urban areas). A secondary sensitivity analysis was performed which restricted to controls selected from Olmstead County. Analysis was conducted with R version 4.2.2 (R Project for Statistical Computing). All tests were 2-sided with a threshold of significance of *P* < .05.

## Results

### Characteristics of Incident Cases

There were 450 incident cases of PD from Olmsted County identified from 1991 to 2015 with 9000 controls matched by age and sex. After excluding PD cases and controls for incomplete residency data prior to the index date (6.9% of cases and 39.0% of controls), there were 419 individuals with PD (median [IQR] age at diagnosis, 73 [65-80] years; 257 male [61.3%]) and 5113 controls (median [IQR] age at index, 72 [65-79] years; 3043 male [59.5%]) included for analysis (Table 1). Although all PD cases had an Olmstead County address at the time of symptom onset, address history data revealed PD cases with previous addresses in 22 of the 27 counties in the study region. The median (IQR) number of addresses recorded in the medical record was 1 (1-2) and the median (IQR) time lived at these addresses was 18.5 (6.0-43.1) years, suggesting that our population was relatively stable in terms of their mobility.

**Table 1.** Characteristics of Incident Parkinson Disease (PD) Cases and Controls

Characteristics	Individuals, No. (%)	
	PD cases (n = 419)	Controls (n = 5113)
Age at index, median (IQR), y	73 (65-80)	72 (65-79)
Sex		
Female	162 (38.7)	2070 (40.5)
Male	257 (61.3)	3043 (59.5)
Race		
American Indian or Alaskan Native	1 (0.2)	9 (0.2)
Asian	6 (1.4)	54 (1.1)
Black or African American	5 (1.2)	29 (0.6)
Hawaiian or Pacific Islander	1 (0.2)	1 (<0.1)
White	403 (96.2)	4504 (88.1)
Other^a^ or multiple races	5 (0.7)	77 (1.3)
Unknown or did not disclose	0	449 (8.9)
Ethnicity		
Hispanic or Latino	7 (1.7)	46 (0.9)
Not Hispanic or Latino	412 (83)	4621 (90.4)
Unknown or did not disclose	0	446 (8.7)
Median household income, median (IQR), thousands $	63.4 (51.4-83.4)	55.6 (46.2-68.0)
RUCA classification		
Urban	337 (80.4)	1557 (30.5)
Suburban	77 (18.4)	2329 (45.6)
Rural	5 (1.2)	1227 (24.0)
Water source		
Groundwater	363 (86.6)	3913 (76.5)
Surface water	0	56 (1.1)
Private well water	56 (13.4)	1144 (22.4)
Pre-index health care utilization, median (IQR), d	8.4 (4.0-15.1)	0.6 (0.0-4.8)
Distance to nearest golf course, median (IQR), miles	1.72 (1.21-2.27)	1.98 (1.19-4.28)

Abbreviation: RUCA, Rural Urban Commuting Area.

^a^ Other was reported directly in medical record and not otherwise defined.

### Risk of Parkinson Disease

**Figure 2.**
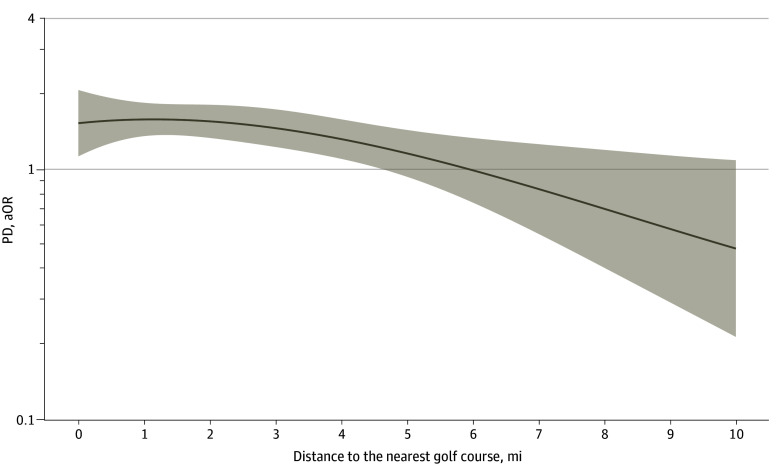
Nonlinear Association Between Odds of Parkinson Disease (PD) and Proximity to a Golf Course Cubic spline fit for the relative risk of Parkinson Disease by proximity to nearest golf course, adjusted for age, sex, race, ethnicity, median household income, rural or urban classification, and health care utilization. aOR indicates adjusted odds ratio; shaded areas represent 95% CIs.

The proximity to the nearest golf course prior to the index date was a median (IQR) distance of 1.72 (1.21-2.27) miles among individuals with PD and 1.98 (1.19-4.28) miles among controls (Wilcoxon rank-sum, *P* < .001). When modeled linearly, the odds for PD decreased by 9% (aOR, 0.91; 95% CI, 0.85-0.98) for every 1-mile increase in distance from a golf course up to 18 miles. The nonlinear association between odds of PD and proximity to a golf course is shown in Figure 2. The odds of PD were relatively constant within close proximity to a golf course and decreased linearly as distance increased; individuals living farther from a golf course had reduced odds of PD, decreasing relative to the distance from the nearest golf course. This was characterized by using linear splines with a regression knot optimized at 3 miles. The odds of PD were not associated with distance from the nearest golf course for those with a pre-index address within 3 miles (per-mile increase: aOR, 0.98; 95% CI, 0.84-1.11) whereas for those farther than 3 miles from a golf course, each additional mile farther from a golf course reduced the odds of PD by 13% (per-mile increase: aOR, 0.87; 95% CI, 0.77-0.98). A likelihood ratio test comparing the spline model with a linear model favored the nonlinear spline for modeling risk of PD (*P* < .001). There was also positive association between living in proximity to golf course and risk of PD when proximity was modeled as a categorical variable, wherein living within 1 mile of a golf course was associated with 126% increased odds of PD compared with those living farther than 6 miles away from a golf course (aOR, 2.26; 95% CI, 1.09-4.70) (Table 2). There was a modest dose response wherein the odds of PD increased by 198% at 1 to 2 miles (aOR, 2.98; 95% CI, 1.46-6.06), 121% at 2 to 3 miles (aOR, 2.21; 95% CI, 1.06-4.59), and 92% at 3 to 6 miles (aOR, 1.92; 95% CI, 0.91-4.04) when compared with those living farther than 6 miles away. Our fully adjusted model demonstrated good model fit (Hosmer-Lemeshow *P* = .12) and very good calibration (Brier score = 0.060) (eFigure 3 in Supplement 1). As a sensitivity analysis, we also repeated our primary analysis after stratifying by RUCA and found that the association between proximity to golf course and PD were stronger in urban areas (eTable 1 in Supplement 1). In our secondary sensitivity analysis, in which we used controls selected from Olmstead County, we found that results remained consistent (eTables 2-3 in Supplement 1).

**Table 2.** Association Between Proximity to Golf Courses and Risk of Parkinson Disease (PD) Compared With Those Living Farthest Away in the 27-County Rochester Epidemiology Project Study Region

Proximity to golf course, miles	All patients, No. (%)^a^		Adjusted for patient demographics^b^		Further adjusted for region characteristics^b^		Further adjusted for vulnerable groundwater^c^	
	PD (n = 419)	Controls (n = 5113)	aOR (95% CI)	*P* value	aOR (95% CI)	*P* value	aOR (95% CI)	*P* value
**Categorical**								
>6	9 (1.1)	807 (98.9)	1 [Reference]	NA	1 [Reference]	NA	1 [Reference]	NA
<1	78 (7.3)	987 (92.7)	7.23 (3.60-14.51)	<.001	2.26 (1.09-4.70)	.03	2.43 (1.16-5.09)	.02
1-2	207 (11.4)	1605 (88.6)	12.38 (6.31-24.27)	<.001	2.98 (1.46-6.06)	.003	2.62 (1.28-5.39)	.01
2-3	82 (9.3)	801 (90.7)	9.53 (4.75-19.12)	<.001	2.21 (1.06-4.59)	.03	2.06 (0.97-4.35)	.06
3-6	43 (4.5)	913 (95.5)	4.38 (2.12-9.04)	<.001	1.92 (0.91-4.04)	.09	2.01 (0.96-4.21)	.07
**Cubic splines**								
0-3^d^	367 (9.8)	3393 (90.2)	1.05 (0.92-1.19)	.49	0.98 (0.84-1.11)	.63	0.97 (0.77-1.22)	.79
>3^e^	52 (2.9)	1720 (97.1)	0.62 (0.54-0.71)	<.001	0.87 (0.77-0.98)	.03	0.87 (0.77-0.99)	.03

Abbreviations: aOR, adjusted odds ratio; NA, not applicable.

^a^ Percentages were calculated row-wise.

^b^ Demographic variables included age, sex, race, and ethnicity. Region characteristics included median household income, Rural Urban Commuting Area classification, and health care utilization.

^c^ Excludes populations outside of Minnesota.

^d^ aORs for 0-3 miles represent the change in odds for PD per 1-mile increase in distance to a golf course within 3 miles.

^e^ aORs for more than 3 miles represent the change in odds for PD per 1-mile increase in distance to a golf course beyond 3 miles.

Individuals getting their tap water from groundwater water service areas with a golf course had nearly doubled odds of PD compared with individuals getting tap water from groundwater water service areas without golf courses (aOR, 1.96; 95% CI, 1.20-3.23) and 49% greater odds of PD compared with individuals getting drinking water from private wells (aOR, 1.49; 95% CI, 1.05-2.13) (Table 3). Our analysis using data on groundwater vulnerability revealed that individuals getting their tap water from water service areas with a golf course in vulnerable groundwater regions had 82% greater odds of PD compared with those in nonvulnerable water service areas with a golf course (aOR, 1.82; 95% CI, 1.09-3.03) and 92% greater odds of PD compared with individuals living in water service areas without a golf course (aOR, 1.92; 95% CI, 1.06-3.45) (Table 3). Additionally, after adding an adjustment for proximity to golf course (as a categorical variable), individuals in vulnerable water service areas had nearly twice the odds for PD compared with individuals getting water from nonvulnerable service areas (aOR, 1.99; 95% CI, 1.30-3.04; *P* = .001). Similar results were found when adjusting for proximity to golf course as linear splines (vulnerable water: aOR, 2.02; 95% CI, 1.33-3.09; *P* = .001). Finally, our analysis of municipal wells revealed no association between PD risk and living in water service areas with a shallow municipal well (aOR, 0.63; 95% CI, 0.24-1.64) or with a municipal well on a golf course (aOR, 0.56; 95% CI, 0.21-1.50) (eTable 4 in Supplement 1).

**Table 3.** Association Between Living Within a Water Service Area With and Without Golf Courses on Vulnerable and Nonvulnerable Groundwater Regions and Risk of Parkinson Disease (PD) in Southern Minnesota

Water service area type	All patients, No. (%)^a^^,^^b^		Adjusted for patient demographics^c^^,^^b^		Further adjusted for region characteristics^c^^,^^b^	
	PD patients (n = 418)	Controls (n = 3996)	aOR (95% CI)	*P* value	aOR (95% CI)	*P* value
Groundwater with golf course^d^	342 (13.3)	2227 (86.7)	1 [Reference]	NA	1 [Reference]	NA
Groundwater without golf course	20 (2.1)	948 (97.9)	0.13 (0.08-0.20)	<.001	0.51 (0.31-0.83)	.01
Private well	56 (6.4)	821 (93.6)	0.41 (0.31-0.55)	<.001	0.67 (0.47-0.95)	.02
Groundwater vulnerability						
Total, No.	362	3175	NA	NA	NA	NA
Vulnerable groundwater with golf course	319 (16.4)	1629 (83.6)	1 [Reference]	NA	1 [Reference]	NA
Vulnerable groundwater with no golf course	14 (2.8)	484 (97.2)	0.14 (0.08-0.23)	<.001	0.74 (0.43-1.27)	.28
Nonvulnerable groundwater with golf course	23 (3.7)	598 (96.3)	0.18 (0.12-0.29)	<.001	0.55 (0.33-0.92)	.02
Nonvulnerable groundwater with no golf course	6 (1.3)	464 (98.7)	0.10 (0.06-0.16)	<.001	0.52 (0.29-0.94)	.03

Abbreviations: aOR, adjusted odds ratio; NA, not applicable.

^a^ Percentages were calculated row-wise.

^b^ Excludes populations outside of Minnesota.

^c^ Demographics included age, sex, race, and ethnicity. Region characteristics included median household income, Rural Urban Commuting Area classification, and health care utilization.

^d^ aORs are reported using the exposed group as the reference in this table and reported for the exposed group in the text (inverse).

## Discussion

In this population-based case-control study, living close to a golf course was associated with an increased risk of developing PD. We observed that risk of developing PD was greatest for those living within 1 to 3 miles of a golf course and that the risk of PD generally decreased with increasing distance from a golf course. We also found that individuals getting their drinking water from water service areas with a golf course had nearly double the odds of PD compared with individuals getting drinking water from water service areas without a golf course. Additionally, the largest effect sizes were for the association of living within a water service area with a golf course in a vulnerable groundwater region.

For years pesticides including organophosphates,^26^ chlorpyrifos,^27^ methylchlorophenoxypropionic acid (MCPP),^28^ 2,4-dichlorophenoxyacetic acid (2,4-D),^28^ maneb,^28^ and organochlorines,^29^ known to be associated with the development of PD, have been used to treat golf courses. Some studies have identified a link between golf courses and increased risk of adverse health outcomes.^26,30,31^ Pesticides such as paraquat and rotenone have been shown to induce Parkinson-like neurodegeneration in the substantia nigra, primarily through mechanisms involving oxidative stress, mitochondrial dysfunction, and dopaminergic neuron apoptosis.^10,32^ However, despite the biological plausibility, very few studies have explored the role of pesticide exposure from golf courses on risk of PD. One study of golf course superintendents found a pattern of pesticide related cancers with a small portion of participants (2 individuals) developing PD.^30^ Another report provided anecdotal evidence of the possible role of golf courses in the development of PD, finding that 19 of 26 patients with PD in a study cohort lived within 2 miles of a golf course.^15^

In our study, after adjusting for socioeconomic and demographic characteristics, the risk of PD was greatest near golf courses. However, there was no difference in PD risk within 3 miles and decreasing levels of risk beyond 3 miles. One possible explanation for the lack of an association within 3 miles was a possible ceiling effect at the higher levels of exposure. Another possible explanation was that exposure may occur through the consumption of a shared, contaminated, groundwater resource in a water service area. Groundwater collected from municipal wells is sent to a water tower where it is treated, pressurized, stored, and distributed to all the residents within the water service area. In our study, 90% of individuals living within 3 miles of a golf course also lived within the boundaries of a water service area serviced with groundwater. Thus, individuals living within the same water service area usually rely on a shared groundwater resource and would therefore receive the same exposure. Nevertheless, we acknowledge that the complexity of the water distribution process varies from city to city and therefore it is possible that not all individuals within the same water service area share the same water resource (eg, in the case of a water service area with multiple water towers). Our study area reflects rural, suburban, and relatively slow-growing major metropolitan cores; thus, we expect the water distribution process to be less complex compared with that of faster growing cities.

Several studies have provided evidence of the ability for pesticides applied to golf courses to leach into the ground and contaminate drinking water supplies.^16,17,33,34^ For instance, 1 study^16^ found that the groundwater under 4 different golf courses in Cape Cod was contaminated with 7 different pesticides, including chlorpyrifos and 2,4-D among others. In this study, 1 pesticide was present in the drinking water at levels more than 200 times greater than the health guidance level. In our study, 77.3% of our patient population (86.6% of cases and 76.5% of controls) lived in water service areas that relied on groundwater resources.

Airborne exposure to pesticides may also drive the relationship between PD risk and proximity to golf courses. In our study, we found that the association between proximity to golf course and PD remained (for those living within 1 to 2 miles) after adjusting for groundwater vulnerability. Moreover, we found larger effect sizes for the association between distance to golf course and PD risk in the urban areas, and thus we speculate that greater city density surrounding golf courses in urban areas may lead to higher levels of airborne pollutant exposure for the nearby residences. Taken together, our study complements, and expands on, the limited research on golf courses as a risk factor for PD and further suggests that both vulnerable drinking water and airborne pollutant exposure may contribute to risk for developing PD near golf courses. Public health policies to reduce the risk of groundwater contamination and airborne exposure from pesticides on golf courses may help reduce risk of PD in nearby neighborhoods.

### Strengths and Limitations

Our study has several strengths. First, we used population-based incidence data, which allows us to better answer questions of PD etiology. Second, rather than relying on *ICD* codes alone, all identified cases were screened by a movement disorder specialist to confirm the onset and diagnosis of PD. Third, our study used address-level data to assign exposure, which provides more accurate distance-to-exposure values compared with studies using less precise location information (eg, zip code centers). Fourth, we digitized and screened our golf course data manually to confirm the correct placement of the golf course boundaries in 2013. Fifth, we integrated data on water service areas, which enabled us to determine whether an individual received drinking water from a groundwater resource, surface water, or a private well.

Our study also has limitations. Our population-based dataset had a limited geographical extent. However, the REP captures data from patients for all health systems within our study area, making it a comprehensive population-based dataset.^20^ Our study was limited in that the population is predominantly White given the demographics of the study region and therefore might limit the generalizability of our results. However, our REP data well replicates other PD-exposure relationships found in diverse cohorts such as the US Medicare population.^1^ Additionally, we acknowledge that requiring PD cases to have an Olmsted County residence at the time of diagnosis is a limitation in our study design. Despite this constraint, our sensitivity analysis, which used controls selected from Olmstead County, provided consistent results. We did not have information on occupational history, thus results may be vulnerable to exposure misclassification errors (ie, for patients who spend more time at locations other than their home address). Our study did not consider other relevant PD risk factors (eg, head trauma, genetic predisposition). Due to the long prodromal period of PD,^35^ we obtained data on golf course locations for the earliest possible year of data available (2013). Although this year of exposure data provided a minimal exposure window, we speculate that most of the golf courses identified in our study existed for many years prior to 2013. Notably, given the long prodromal period of PD, our study uses distance as a proxy to estimate exposure to pesticides occurring many decades ago, which may not reflect the same pesticides applied on these golf courses in recent years. Nevertheless, even if golf courses were present decades earlier, our results were based on home address information 2 to 3 years prior to symptom onset, which does not capture the complete prodromal period. Our population was relatively stable, living at their address for approximately 6 to over 43 years. We were unable to extend address history back further than 3 years because residency data becomes more incomplete the further we move back in time due to lack of interaction with the health care system.

## Conclusions

This population-based case-control study provides evidence in support of an association between living within proximity to golf courses and the risk of developing PD. Shorter distances from golf courses were associated with an increased risk of PD compared with those living farther away. Associations with the largest increase in odds was found in individuals living within water service areas with a golf course and in vulnerable ground water regions.

## Notes

Supplement 1. **eTable 1.** Sensitivity Analysis for RUCA **eTable 2.** Differences Between Olmsted and Non-Olmsted Controls **eTable 3.** Primary Analysis With Olmsted Controls **eTable 4.** Association Between Minimum Municipal Well Depth Within a Water Service Area (WSA) and Risk of Parkinson Disease and Association Between Presence of Municipal Well on a Golf Course and Risk of Parkinson Disease in the 27-County Rochester Epidemiology Project Study Area **eFigure 1.** A Contour Map Showing the Distance in Miles to the Edge of the Nearest Golf Course in Southeast Minnesota and Northwest Wisconsin **eFigure 2.** A Map of Water Service Areas and Golf Courses on Vulnerable and Non-Vulnerable Ground Water **eFigure 3.** Standard Calibration Plot for Predicting Risk of PD Using Categorical Proximity to Golf Course, Patient Demographics, and Region Characteristics (Brier Score 0.060)

Supplement 2.
Data Sharing Statement
