# Supplementary material for: Proximity to Golf Courses and Risk of Parkinson Disease
Source: JAMA Netw Open. 2025 May 8;8(5):e259198. doi: 10.1001/jamanetworkopen.2025.9198 (PMC12062912; doi:10.1001/jamanetworkopen.2025.9198)
Supplement: Supplement 1. — eTable 1. Sensitivity Analysis for RUCA eTable 2. Differences Between Olmsted and Non-Olmsted Controls eTable 3. Primary Analysis With Olmsted Controls eTable 4. Association Between Minimum Municipal Well Depth Within a Water Service Area (WSA) and Risk of Parkinson Disease and Association Between Presence of Municipal Well on a Golf Course and Risk of Parkinson Disease in the 27-County Rochester Epidemiology Project Study Area eFigure 1. A Contour Map Showing the Distance in Miles to the Edge of the Nearest Golf Course in Southeast Minnesota and Northwest Wisconsin eFigure 2. A Map of Water Service Areas and Golf Courses on Vulnerable and Non-Vulnerable Ground Water eFigure 3. Standard Calibration Plot for Predicting Risk of PD Using Categorical Proximity to Golf Course, Patient Demographics, and Region Characteristics (Brier Score 0.060) [file jamanetwopen-e259198-s001.pdf]

Supplemental Online Content

Krzyzanowski B, Mullan AF, Dorsey ER, et al. Proximity to golf courses and risk of Parkinson disease. *JAMA Netw Open.* 2025;8(5):e259198. doi:10.1001/jamanetworkopen.2025.9198

eTable 1. Sensitivity Analysis for RUCA

eTable 2. Differences Between Olmsted and Non-Olmsted Controls

eTable 3. Primary Analysis With Olmsted Controls

eTable 4. Association Between Minimum Municipal Well Depth Within a Water Service Area (WSA) and Risk of Parkinson Disease and Association Between Presence of Municipal Well on a Golf Course and Risk of Parkinson Disease in the 27-County Rochester Epidemiology Project Study Area

eFigure 1. A Contour Map Showing the Distance in Miles to the Edge of the Nearest Golf Course in Southeast Minnesota and Northwest Wisconsin

eFigure 2. A Map of Water Service Areas and Golf Courses on Vulnerable and Non-Vulnerable Ground Water

eFigure 3. Standard Calibration Plot for Predicting Risk of PD Using Categorical Proximity to Golf Course, Patient Demographics, and Region Characteristics (Brier Score 0.060)

This supplemental material has been provided by the authors to give readers additional information about their work.

© 2025 Krzyzanowski B et al. *JAMA Network Open*.

eTable 1: Sensitivity analysis for RUCA

**Categorical**	**Individuals in Urban Areas (RUCA = 1)**		**Further Adjusted for Region Characteristics[2]**	
**Proximity to Golf Course**	**PD Patients** (N = 337)	**Controls** (N = 1557)	**aOR** (95% CI)	**P-Value**
Less than 1 mile	60 (18.0%)	274 (82.0%)	2.71 (1.41, 5.22)	0.003
1-2 miles	188 (20.7%)	719 (79.3%)	3.31 (1.80, 6.09)	< 0.001
2-3 miles	76 (17.8%)	350 (82.2%)	2.54 (1.34, 4.81)	0.004
More than 3 miles	13 (5.7%)	214 (94.3%)	Reference	---
**Cubic Splines**	**All Patients[1]**		**Further Adjusted for Region Characteristics[2]**	
**Proximity to Golf Course**	**PD Patients** (N = 337)	**Controls** (N = 1557)	**aOR** (95% CI), per ½ mile	**P-Value**
0-3 miles	324 (19.4%)	1343 (80.6%)	0.99 (0.91, 1.09)	0.94
More than 3 miles	13 (5.7%)	214 (94.3%)	0.49 (0.30, 0.79)	0.003

[1]Percentages were calculated row-wise
[2]Demographics included age, sex, race, and ethnicity. Region characteristics included median household income, and healthcare utilization

© 2025 Krzyzanowski B et al. *JAMA Network Open*.

eTable 2: Differences between Olmsted and non-Olmsted controls

	**Olmsted Controls** (N = 1727)	**Non-Olmsted Controls** (N = 3386)
Age at index, years		
Median (Q1, Q3)	71 (63, 78)	73 (66, 80)
Sex, n (%)		
Female	702 (40.6%)	1368 (40.4%)
Male	1025 (59.4%)	2018 (59.6%)
Race, n (%)		
American Indian/Alaskan Native	4 (0.2%)	5 (0.1%)
Asian	26 (1.5%)	28 (0.8%)
Black/African American	27 (1.6%)	2 (0.1%)
Hawaiian/Pacific Islander	1 (0.1%)	0 (0%)
White	1406 (81.4%)	3098 (91.5%)
Other/Mixed Race	21 (1.2%)	46 (1.4%)
Unknown/Did not disclose	242 (14.0%)	207 (6.1%)
Ethnicity, n (%)		
Hispanic or Latino	18 (1.0%)	28 (0.8%)
Not Hispanic or Latino	1468 (85.0%)	3153 (93.1%)
Unknown/Did not disclose	241 (14.0%)	205 (6.1%)
Median household income, thousands of dollars		
Median (Q1, Q3)	61.3 (48.7, 78.5)	54.0 (45.8, 63.3)
RUCA classification, n (%)		
Urban	1157 (67.0%)	400 (11.8%)
Suburban	519 (30.1%)	1810 (53.5%)
Rural	51 (3.0%)	1176 (34.7%)
Water source, n (%)		
Ground water	1440 (83.4%)	2473 (73.0%)
Surface water	0 (0%)	56 (1.7%)
Private well water	287 (16.6%)	857 (25.3%)
Pre-index healthcare utilization, days		
Median (Q1, Q3)	2.6 (0.0, 8.8)	0.2 (0.0, 3.0)
Any utilization within 5 years, n (%)	1148 (66.5%)	1842 (54.4%)
Distance to nearest golf course, miles		
Median (Q1, Q3)	1.75 (1.17, 2.58)	2.30 (1.20, 5.34)

© 2025 Krzyzanowski B et al. *JAMA Network Open*.

eTable 3: Primary analysis with Olmsted controls

Proximity to Golf Course	All Patients			PD versus Olmsted Controls		PD versus non-Olmsted Controls	
	**PD Patients** (N=419)	**Olmsted Controls** (N=1727)	**Non-Olmsted Controls** (N=3386)	**aOR** (95% CI)	**P-Value**	**aOR** (95% CI)	**P-Value**
Less than 1 mile	78 (7.3%)	334 (31.4%)	653 (61.3%)	2.33 (1.10, 4.94)	0.027	2.78 (1.26, 6.12)	0.011
1-2 miles	207 (11.4%)	737 (40.7%)	868 (47.9%)	2.90 (1.41, 5.97)	0.004	4.27 (2.04, 8.91)	< 0.001
2-3 miles	82 (9.3%)	309 (35.0%)	492 (55.7%)	2.93 (1.13, 5.06)	0.022	2.31 (1.05, 5.06)	0.037
3-6 miles	43 (4.5%)	265 (27.7%)	648 (67.8%)	1.60 (0.74, 3.50)	0.24	1.43 (0.64, 3.18)	0.38
More than 6 miles	9 (1.1%)	82 (10.0%)	725 (88.8%)	Reference	---	Reference	---

[1]aORs were adjusted for patient demographics (age, sex, race, and ethnicity), median household income, RUCA classification, and healthcare utilization.

© 2025 Krzyzanowski B et al. *JAMA Network Open*.

eTable 4. Association between minimum municipal well depth within a water service area (WSA) and risk of Parkinson's Disease and association between presence of municipal well on a golf course and risk of Parkinson's Disease in the 27-county Rochester Epidemiology Project study area.

Minimum Well Depth	All Patients[1]		All WSAs		WSAs with a golf course	
	PD Patients (N = 115)	Controls (N = 1526)	aOR[2] (95% CI)	P-Value	aOR (95% CI)	P-Value
Shallow, <100ft	6 (10.2%)	53 (89.9%)	Reference	---	Reference	---
Deep, >100ft	109 (6.9%)	1,473 (93.1%)	0.63 (0.24, 1.64)	0.34	0.56 (0.21, 1.50)	0.25

Municipal Well Location	All Patients[1]		All WSAs		WSA with vulnerable groundwater	
	PD Patients (N = 239)	Controls (N = 3448)	aOR[2] (95% CI)	P-Value	aOR[2] (95% CI)	P-Value
No wells on a golf course	115 (5.9%)	1839 (94.1%)	Reference	---	Reference	---
At least one well on a golf course	124 (7.2%)	1609 (92.8%)	1.25 (0.94, 1.66)	0.12	1.13 (0.84, 1.52)	0.41

[1] Percentages were calculated row-wise

[2] Odds ratios were adjusted for demographics (age, sex, race, and ethnicity) and region characteristics (median household income, RUCA classification, and healthcare utilization).

© 2025 Krzyzanowski B et al. *JAMA Network Open*.

eFigure 1: A contour map showing the distance in miles to the edge of the nearest golf course in Southeast Minnesota and Northwest Wisconsin.

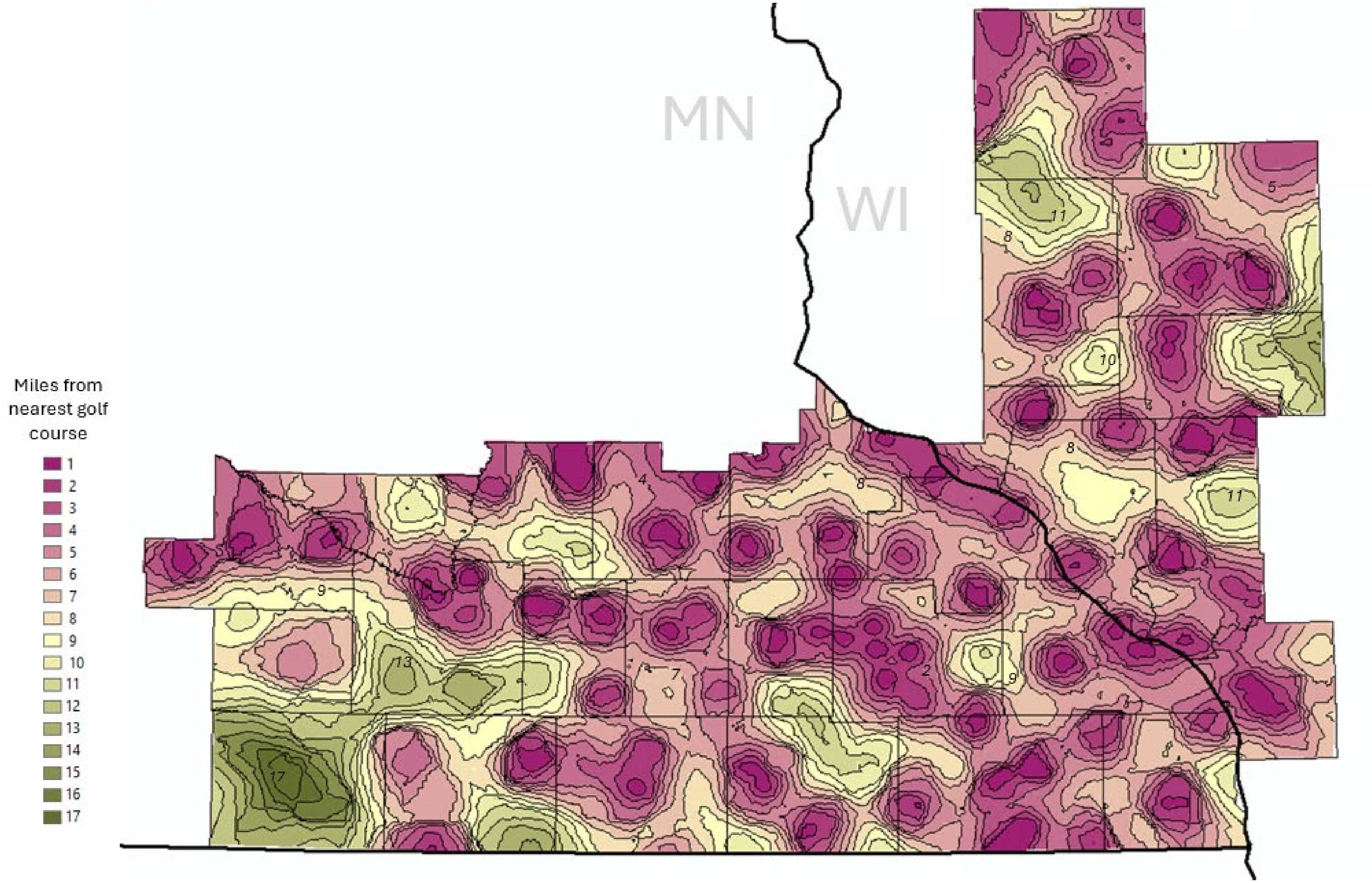

© 2025 Krzyzanowski B et al. *JAMA Network Open*.

eFigure 2: A map of water service areas and golf courses on vulnerable and non-vulnerable ground water.

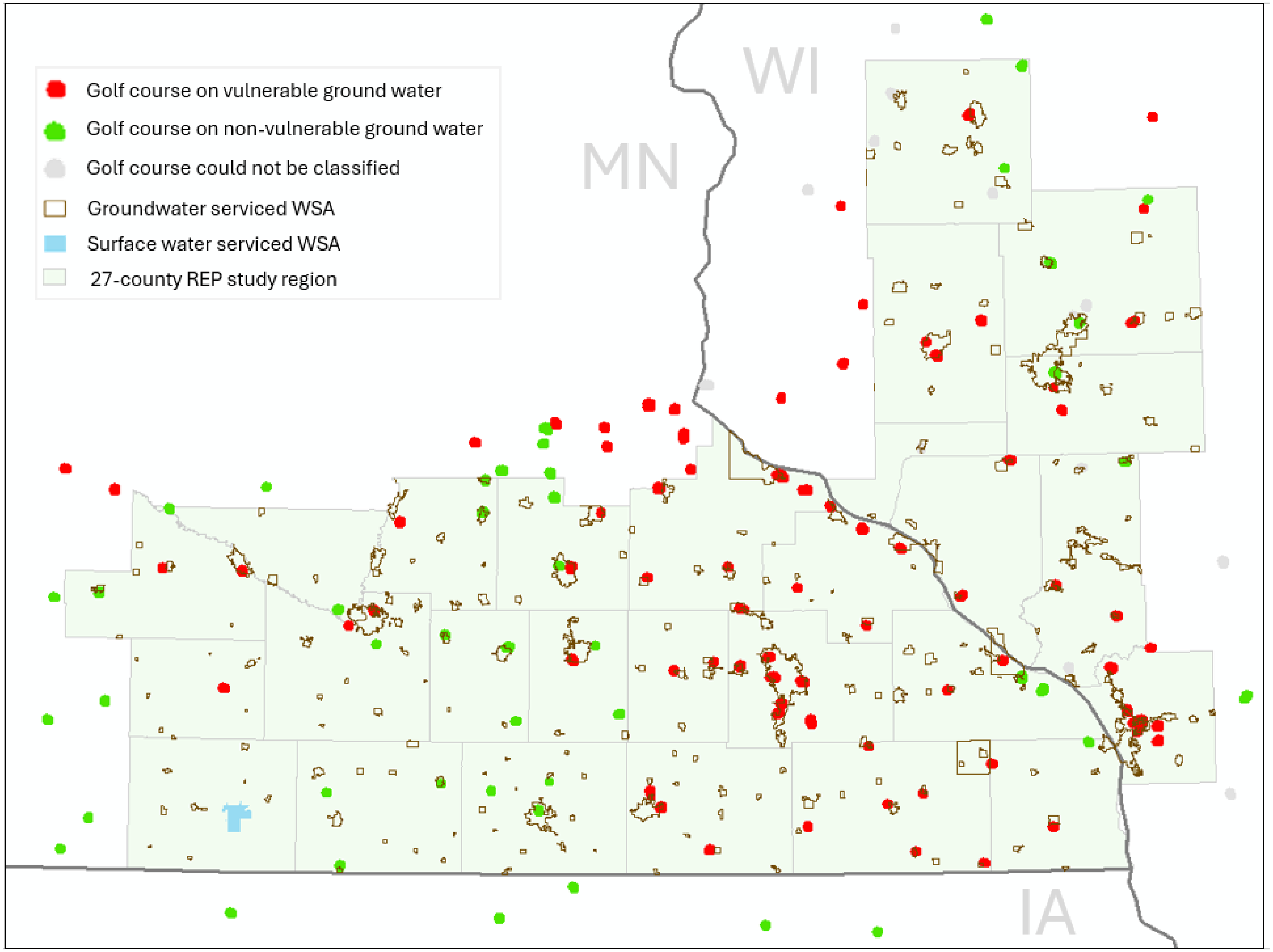

© 2025 Krzyzanowski B et al. *JAMA Network Open*.

eFigure 3. Standard calibration plot for predicting risk of PD using categorical proximity to golf course, patient demographics, and region characteristics (Brier score 0.060)

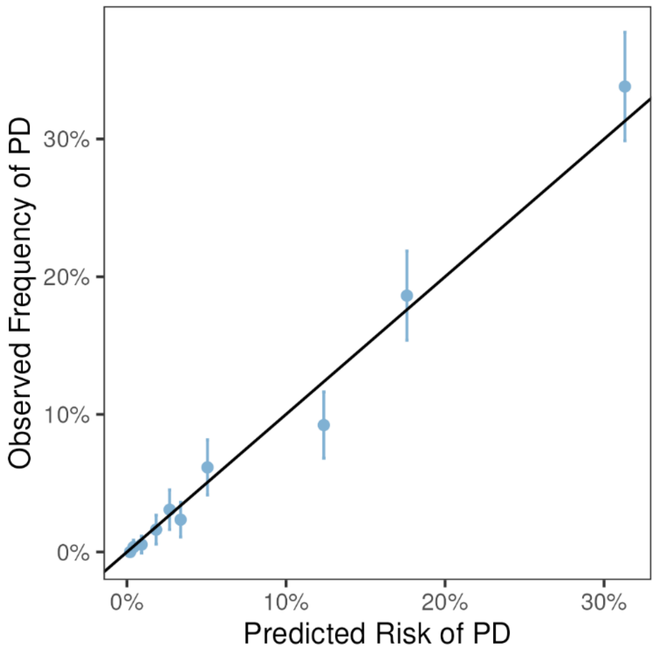

© 2025 Krzyzanowski B et al. *JAMA Network Open*.
