# Supplementary material for: Proximity to Golf Courses and Risk of Parkinson Disease
Source: JAMA Netw Open. 2025 May 8;8(5):e259198. doi: 10.1001/jamanetworkopen.2025.9198 (PMC12062912; doi:10.1001/jamanetworkopen.2025.9198)
Supplement: Supplement 2. — Data Sharing Statement [file jamanetwopen-e259198-s002.pdf]

Data Sharing Statement

Krzyzanowski. Proximity to Golf Courses and Risk of Parkinson Disease. *JAMA Netw Open*. Published May 08, 2025. doi:10.1001/jamanetworkopen.2025.9198

Data
Data available: No

Additional Information
Explanation for why data not available: This data contains protected health information and cannot be shared.
